# Supplementary material for: Splice-Junction-Based Mapping of Alternative Isoforms in the Human Proteome
Source: Cell Rep. Author manuscript; Available in PMC 2020 Jan 15. (PMC6961840; doi:10.1016/j.celrep.2019.11.026)
Supplement: 3 [file NIHMS1546469-supplement-3.zip › DF2/PXD0010154/Pancreas-110-Q9H4A3-QSTQGVSQVAPAEPVAVAQTQATQPTT.pdf]

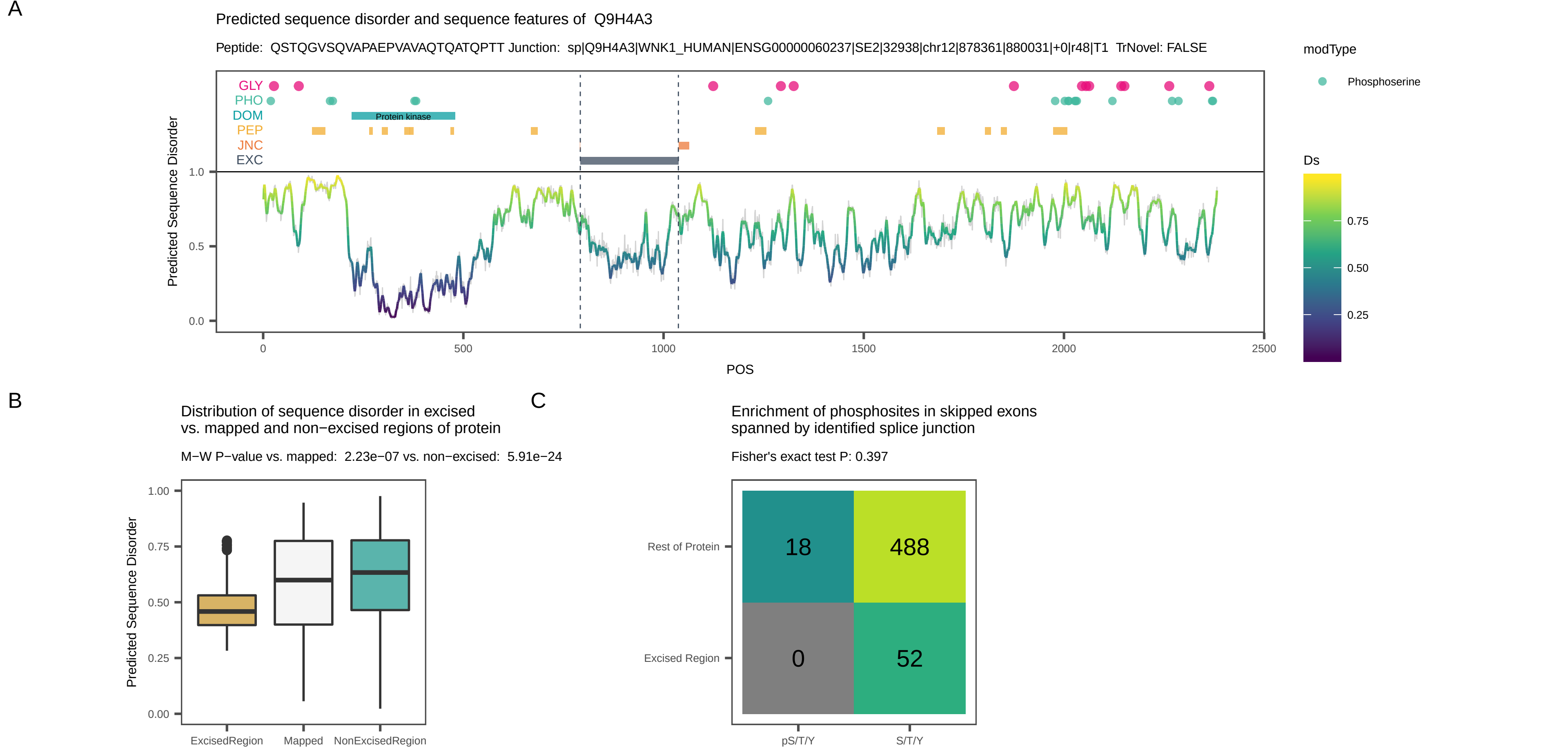

A
Predicted sequence disorder and sequence features of Q9H4A3
Peptide: QSTQGVSQVAPAEPVAVAQTQATQPTT Junction: sp|Q9H4A3|WNK1_HUMAN|ENSG00000060237|SE2|32938|chr12|878361|880031|+0|r48|T1 TrNovel: FALSE
GLY
PHO
DOM
PEP
JNC
EXC
Protein kinase
Predicted Sequence Disorder
1.0
0.5
0.0
0
500
1000
1500
2000
2500
POS
modType
Phosphoserine
Ds
0.75
0.50
0.25
B
Distribution of sequence disorder in excised vs. mapped and non−excised regions of protein
M−W P−value vs. mapped: 2.23e−07 vs. non−excised: 5.91e−24
Predicted Sequence Disorder
1.00
0.75
0.50
0.25
0.00
ExcisedRegion
Mapped
NonExcisedRegion
C
Enrichment of phosphosites in skipped exons spanned by identified splice junction
Fisher's exact test P: 0.397
Rest of Protein
Excised Region
18
488
0
52
pS/T/Y
S/T/Y
